# Responsibility of citizens in food safety social co-governance in the context of China

**DOI:** 10.3389/fpubh.2022.962629

**Published:** 2022-08-02

**Authors:** Linhai Wu, Ke Qin, Xiujuan Chen

**Affiliations:** ^1^Institute for Food Safety Risk Management, School of Business, Jiangnan University, Wuxi, China; ^2^School of Business, Jiangnan University, Wuxi, China

**Keywords:** food safety social co-governance, responsible actor, citizen responsibility, mixed logit, best-worst scaling (BWS)

## Abstract

**Objective:**

This study aims to identify all actors that hold some responsibility for ensuring food safety based on the complete food supply chain in the context of China's current circumstances.

**Methods:**

The study was conducted among citizens in Wuxi, Jiangsu, China. All citizens fully understood the purpose of the study and voluntarily agreed to participate. From December 10 to 14, 2020, a total of 398 valid samples were collected by the researchers using a structured questionnaire. Survey data were assessed using best–worst scaling and a mixed logit model from the perspective of citizen responsibility.

**Results:**

In descending order, responsibility for ensuring food safety goes from food producers and traders (including producers, distributors, and retailers) to the government, social organizations, news media, and finally to citizens. Food producers and traders are the actors who should take the greatest responsibility, whereas citizens bear the least responsibility.

**Conclusion:**

The responsibility of citizens in food safety co-governance should be recognized but it should not be arbitrarily extended.

## Introduction

Food safety is a major public issue facing all countries around the world (1, 2), and it has been identified as a public health priority by the World Health Organization (3). Numerous studies and practical experiences have demonstrated that as industrialization proceeds, production technologies become more and more complex, and the food industry structure become increasingly advanced. In this context, even a small food safety risk has the potential to threaten consumer dietary health throughout the food supply chain due to the butterfly effect (4, 5). Therefore, government or market failures, either alone or combined, are difficult to avoid by relying solely on these actors (acting alone or together) to manage food safety risks. Thus, the traditional governance model has been unable to effectively meet society's consumption needs regarding food safety (6, 7). Ensuring food safety is therefore the common responsibility of all stakeholders, which requires each of them to perform their respective functions (8). The phenomenon of social co-governance first emerged in the 1960s and 1970s and has since developed as a new governance model in Western countries. It evolved in response to changes in the relationship among government, market, and society (9). In brief, a food safety social co-governance system enables all stakeholders, including the government, enterprises (market), social organizations, and citizens, to fulfill their respective responsibilities in accordance with the law, participate in the formulation and revision of food safety laws, regulations, and rules in different manners, and coordinate and cooperate with each other, thereby collectively ensuring food safety with low governance costs in an open, transparent, and flexible manner (10). Although China has different national conditions from those in Western countries, the Chinese government began to reform the country's food safety governance model in 2013, especially in 2015 and through the revised Food Safety Law, which identified social co-governance as the basic mechanism for managing food safety risks (11). However, at present, there are still serious deficiencies in the construction of a food safety social co-governance system in China. One such important aspect is that most citizens regard themselves simply as food consumers. They seldom actively participate in the supervision of food production and distribution and do not actively engage with or lack channels through which to participate in the formulation of government food policies (12). Most consumers even keep silent when harmed by defective food products. However, individuals are the best judges of their own actions (13). Because food safety is a major public issue related to individual health and social stability, each citizen can be considered a natural and unique participant in the food safety social co-governance system, wherein citizens have unique functions that differ from those of government, market, and social organizations. A food safety social co-governance system cannot exist without citizens' active and broad participation. Therefore, this study includes citizen responsibility in the food safety social co-governance system to investigate the responsibilities of all key responsible actors, including citizens, in ensuring food safety in the context of China, and objectively evaluates citizens' responsibilities in the food safety co-governance system, with a view to ensuring that citizens are willing and able to participate in food safety co-governance, which is the long-term solution to ensuring food safety.

Ensuring food safety is one of a government's basic responsibilities (14). Government responsibility has become an important symbol of a democratic political system. However, the high cost of government regulation provides space for opportunistic behaviors by producers and traders, resulting in frequent food safety incidents (15). In China, the government is coming under increasing pressure from regulations and public opinion as food safety issues become increasingly complex. Increasingly, all food safety problems are being attributed to inadequate government regulation (16). Therefore, given the increasingly complex food safety risk factors, it is necessary to change the traditional government-based one-way governance structure, rely more on the cooperation of multiple stakeholders, such as the government, enterprises, and the public, and develop good citizenship by activating social forces, mobilizing broad public participation, and increasing citizen engagement (17, 18). Despite the current overall trend of improving food safety in China, food safety risks are highly complicated due to the interconnections of pre-market and market risks and integration of traditional and new risks (19), making decision-making in risk governance complicated for the government. In terms of long-term governance, this responsibility clearly cannot be borne entirely by the government, but should be spread across stakeholders (20). In fact, every citizen has a responsibility for considering food safety along the entire process from farm to fork (21). As food consumers, the public is also a stakeholder and should participate in food safety social co-governance (22). However, there is a conspicuous absence of responsibility among Chinese citizens, including the lack of a public responsibility culture, low participation rate, and difficulty in maintaining enthusiasm for participation (23). Moreover, due to the frequent occurrence of food safety incidents over a long-term period, Chinese citizens now lack confidence and trust in food safety, which has become an important obstacle for them to effectively participate in food safety social co-governance (24). Instead, citizens only tend to pay attention to food safety when a food scandal comes to light (25). In addition, Shang et al. (26) found that most citizens lacked basic knowledge about, had a low willingness to pay for, and took a wait-and-see attitude toward traceable food, which makes it difficult to establish a traceable food market in China. This indifferent attitude also exists in other countries. Mensah et al. (27) found that ordinary citizens in Ghana often turn a blind eye to street food vendors with hygiene problems. Wiatrowski et al. (28) conducted a survey study in Poland and found that most respondents did not care about the hygiene of street food.

In fact, citizenship entails responsibilities. Participation in public affairs is an inherent duty of citizenship (29). However, citizens are overly dependent on the government, lack due understanding of their own responsibilities in public crisis management, and have not had their sense of responsibility and ability developed adequately, resulting in the government being overloaded with responsibilities (30). In terms of food safety risk management in China, consumer protection has always been emphasized in legislation and practice. Although this practice is beyond reproach, it has long neglected the cultivation of a sense of responsibility among consumers, resulting in common unawareness of consumer responsibility (31). However, consumers should not simply be viewed as a vulnerable group to be sympathized with or protected. Consumers' awareness of their responsibilities and abilities regarding food safety will help the government to act in a correct and responsible manner (12). Xue and Zhang (32) found that up to 70% of fatalities due to home-acquired food poisoning in China were caused by improper food handling by household members. Consumers' literacy and abilities in relation to food safety affect their individual and household's food safety (33). Bakhtavoryan et al. (34) found in the case of a 2007 peanut butter recall in the United States that American consumers responded quickly to foodborne disease outbreaks, which motivated companies to improve their food safety emergency management system. Therefore, in addition to the strong government measures, citizens should actively cooperate with the implementation of food safety governance policies and assume their responsibilities to consciously protect public interests in food safety (35).

Citizens can play a critical role in food safety risk management (36). Accordingly, solving the issues of how to mobilize citizens to participate in co-governance and how to reconstruct the food safety governance structure to make citizens an important participant are central for China's efforts to build a food safety social co-governance system (37). The results of previous studies have important implications for the present study. However, this research area is still in its infancy in mainland China. Due to differences in cultural practices and economic development, the conclusions of studies performed outside China may be unable to explain the special problems in food safety risk management in large developing countries like China. The applicability of these conclusions to China needs to be further examined.

In this study, we attempted to incorporate citizens into the food safety social co-governance system, and scientifically identify the key responsible actors, including the government, enterprises, social organizations, and citizens, in the co-governance system. Moreover, we investigated the responsibilities of these key actors for ensuring food safety and objectively evaluated citizens' responsibilities in the food safety co-governance system in the context of China using best–worst scaling (BWS) and a mixed logit model, thereby eliciting the policy implications for enabling citizens to play their role in China, which is an innovative perspective and methodology.

## Materials and methods

### Categories of responsible actors

Many traditional stakeholders exist in food safety. Meanwhile, the continuous development of online trading has seen the emergence of trading platforms and WeChat Moments, etc., as new stakeholders. Nevertheless, as early as 1995, the Commission on Global Governance (CGG) put forward a generally accepted view based on extensive academic research, namely that the government, food producers and traders, social organizations, and citizens are the four fundamental types of responsible actors in the food safety co-governance system in Western countries (38). Other stakeholders can be categorized as one of these types according to their functions. For example, online food trading platforms are essentially retailers. At present, responsible actors in food safety social co-governance system in China have been classified according to different schemes into three, five, or seven types (39–41). Based on China's current conditions and the findings of previous studies, this study suggests that the government, food producers and traders, social organizations, news media, and citizens are the five fundamental types of responsible stakeholders in the country's co-governance system. They can be further divided into seven actors with basically clear boundaries and relatively independent functions. These actors are detailed as follows.

#### Food producers and traders

The most basic element of food safety is production, i.e., all steps of the production process before food reaches consumers' plates. Ensuring food safety thereby requires combined efforts from producers, distributors, and retailers, all of whom are indispensable. These three actors have clear boundaries and are mutually irreplaceable. Together, they constitute the whole process from farm to fork. As the actors most directly involved in ensuring food safety, they should hold primary responsibility for jointly ensuring food safety (42), in keeping with common practice around the world (43). Therefore, in this study, food producers, distributors, and retailers are regarded as three independent actors who share the primary responsibility for food safety, thereby increasing the number of actors from five to seven in this paper.

#### Government

Food safety also relies on governance. As a major public health issue, food safety risks are the shared responsibility of governments around the world (44). In China, food safety has been identified as a major issue in terms of both livelihood and politics and therefore requires coordinated efforts from all levels of government. Of course, governments in different countries have enacted very different systems for managing food safety risks. For example, the Food and Drug Administration, founded in 1906, is the only department of the U.S. government that implements food safety regulations. In China, food safety regulators have undergone dynamic changes since the reform and opening up. Before 2013, multi-department governance had been employed. For example, approximately 10 government departments participated in the management of pork from farm to fork. After two reforms in 2013 and 2019, a regulation system based on three departments, including the Ministry of Agriculture and Rural Affairs, market regulation department, and health department, was developed, with the participation of more than 10 other departments. Therefore, government regulators are more or less substitutable with each other. Given the changes in government regulation of food safety in China, this study regards all such multiple government regulators as a whole in terms of performing regulatory functions on the government's behalf.

#### Social organizations

Social organizations are diverse in form and function. Based on the classification used in Western countries as well as the current situation of China, this study divides social organizations that can play a role in the co-governance system into the following four types. The first type is professional food industry organizations. In China, such organizations include food industry associations, food fermentation associations, etc., which have professional, industry-specific, and self-disciplined functions in food safety co-governance. The second type is food testing organizations. Such organizations have been entrusted with inspecting the quality of food produced by enterprises. Although an entrusted passive behavior, it has higher credibility. Especially in the case of disputes, it plays a greater role than the active inspection of industry and consumer associations (45). The third type is food certification and accreditation organizations. Such organizations investigate and supervise food producers and traders in accordance with the law, and make recommendations to the government or relevant organizations about revoking the qualifications of enterprises that no longer meet the requirements (46). Finally, the fourth type is consumer organizations. Such organizations are non-governmental organizations founded by consumers to communicate with producers and traders in order to maintain their own rights and interests (47). They have wide coverage and great influence, performing an important social function in terms of protecting the rights and interests of consumers (48). It should be pointed out that although these four types of social organizations have different functions and responsibilities, there are no absolute boundaries between them, and they are mutually substitutable to a certain extent. For example, professional food industry organizations can also accept complaints from consumers and carry out some of the same functions as consumer organizations; they may also function as food testing organizations. Therefore, these social organizations are merged into one actor in the co-governance system in this study.

#### News media

News media can take advantage of their strong influence and rapid information dissemination capabilities to alleviate food safety information asymmetry to a certain extent. For example, the melamine-tainted milk powder crisis was widely reported by different types of news media within 4 months of its outbreak in August 2008. The incident's huge scale not only caused a sensation in China, but also attracted extensive attention from the international community. On the one hand, the strong public opinion motivated government regulators to enhance food safety regulation. On the other hand, online public opinion began to be noticed by a wide range of citizens at different levels in China, which initiated the process of mobilizing citizens to participate in food safety risk management. As such, the news media can act as a “vanguard” and “watchmen” in the food safety social co-governance system in China (46, 49). News media can be considered a social organization, and it enjoys an independent legal status. Like professional food industry organizations, media outlets have the function of communicating with the government, food producers and traders, and society, and serve as an important bridge to overcome government and market failures (50). In this study, the media is regarded as an individual actor rather than being included into the category of social organizations. This is not only because the media lacks the professional competencies of food industry organizations, but more importantly, most news media outlets have some attributes of government organizations in the context of China. They have a unique function different from those of any other types of social organizations in the co-governance system. This is an important distinction from Western countries. In Western constitutional theory, the separation of legislative, executive, and judicial powers is an institutional design to restrict government power. The regulatory power of the media is called the fourth power in addition to these three powers (18).

#### Citizens

As indicated in the literature review, citizens capable of independent behavior are not simply food consumers. They not only should assume the responsibility of self-protection, but can also act as the best participant and regulator of food safety. As such, they are an important force in social co-governance, playing an irreplaceable role. In the context of China, a citizen is a person who has citizenship and thus has certain rights as well as obligations. It represents the concept of an individual. However, in this study, citizens included in the co-governance system to assume the corresponding responsibility are a collective concept, representing a collection of individual citizens.

**Figure 1:**
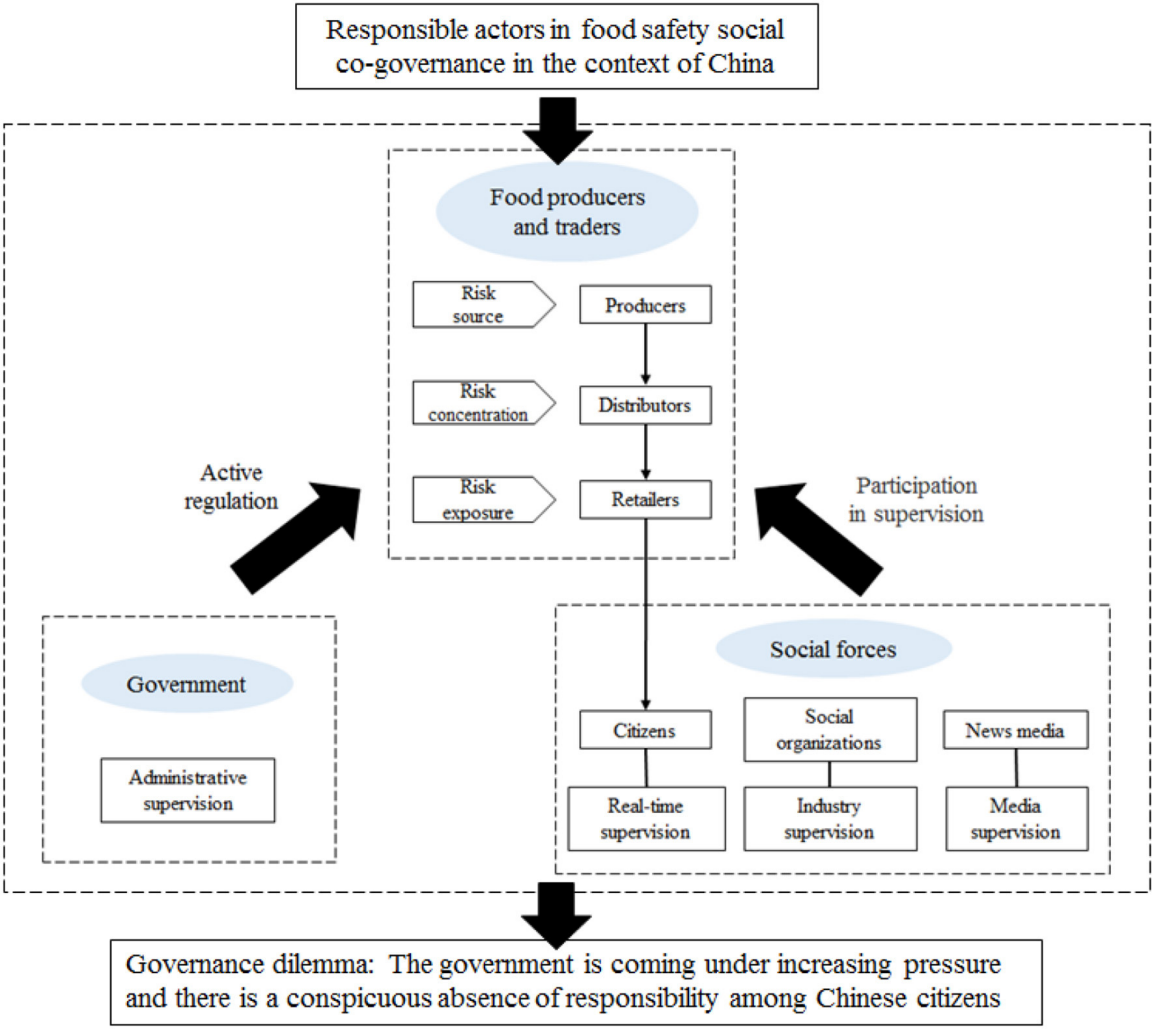
Research framework.

To sum up, this study develops an analytical framework shown in Figure 1 based on the reality of China. This study suggests that the government, food producers and traders, social organizations, news media, and citizens are the five fundamental types of responsible stakeholders in the co-governance system. Moreover, food producers and traders are subdivided into three basic actors, i.e., producers, distributors, and retailers, which have clear boundaries and are not mutually replaceable, together constituting the whole food supply chain from farm to fork. As the actors most directly involved in ensuring food safety, they should hold primary responsibility for jointly ensuring food safety. However, it is far from enough to rely solely on food producers and traders to ensure food safety as it is impossible for them to take responsibility totally on their own initiative. Due to the intertwining of objective factors, such as science and technology, and ecological environment, with human factors, food safety risks are superimposed over one another in different parts of the supply chain, which will induce food safety incidents and aggravate the harm of food safety risks. The management of food safety risks is the common responsibility of all stakeholders, and requires the joint participation of the government and social forces. China is in the midst of drastic social changes. The food safety risks it faces have inherent characteristics different from those of Western countries. The Chinese government is not only faced with the scarcity of governance resources, but also with heavy regulatory tasks. As an important social force, citizens should take the initiative to assume their responsibilities. However, there is in fact a conspicuous absence of responsibility among Chinese citizens. Therefore, this study investigates the responsibilities of key responsible actors in ensuring food safety in the context of China, and objectively evaluates citizens' responsibilities in the food safety co-governance system with a view to providing guidance for the Chinese government to develop institutional arrangements for citizens to play a role in managing food safety risks.

### Sample and data collection

The data for this study were collected by a questionnaire survey. Wuxi in Jiangsu Province has been among the leading large and medium-sized cities in China in terms of economic and social development. In 2020, Wuxi had the highest per capita GDP among all cities in China. Ordinary people generally live in good conditions and show relatively high satisfaction with food safety. This area therefore provides a good foundation for this study. Sample data were collected from a field questionnaire survey in the five administrative districts of Wuxi, including Liangxi, Xishan, Huishan, Binhu, and Xinwu districts. The sample size for each district was proportional to the respective resident population. The survey was carried out among citizens aged over 18 years (hereinafter referred to as respondents) in farmers' markets, chain supermarkets, and food stores with a large flow of customers in each district. Investigators were instructed to select the third person coming into view as a respondent to maximize sample randomness (51). The questionnaires were completed anonymously by respondents at the survey sites and collected once completed. The entire survey process was completed in the period December 10–14, 2020. A total of 398 valid samples were collected.

### Measures

BWS, also known as maximum difference scaling (MaxDiff), is an extension of pairwise comparison (52). As an important method to investigate professional issues, such as psychological values, consumer preferences, and corporate social responsibility (53–55), BWS asks respondents to select the “best” and “worst” items from a set of options based on their personal perceptions. Erdem and Rigby (56) pointed out that respondents' choices of “best” and “worst” items will maximize the difference in their preferences, thereby allowing identification of the set of preferences for a specific option based on group selection of that option by different respondents. Generally speaking, when methods such as the Likert five-point scale are used to elicit preferences, respondents may have ambiguous choices when faced with five different options due to differences in understanding. This problem can be avoided by using BWS. However, measuring multi-level preferences by having respondents identify their choices of best and worst items using BWS can effectively eliminate the measurement error between different preference levels (57). This allows accurate ranking according to respondents' preferences (58), ensuring that conclusions are more in line with reality (59, 60). Accordingly, this study attempts to determine the responsibilities of all stakeholders in the entire food supply chain according to citizens' preferences using BWS in combination with the mixed logit model, thereby better reflecting citizens' perceptions of their own responsibilities.

According to Louviere et al. (61), offering more than five options may fatigue respondents and thus may lead to a preference bias. Therefore, using SSIWeb 7.0 software, the questionnaire was designed so that, first, the seven responsible actors were randomly combined into seven choice groups. Then, a balanced incomplete block design (54) was used to ensure that the options in each choice group satisfy the orthogonality. Each choice group included three actors to choose from. The number of occurrences of each actor in the questionnaire was the same (three times). Respondents were asked to select one of the three actors in each group that should bear the most or the least responsibility. Table 1 is a sample questionnaire designed based on BWS.

**Table 1:** Sample questionnaire designed based on BWS.

**Actor that should**	**Options**	**Actor that should**
**bear the greatest**		**bear the least**
**responsibility**		**responsibility**
□	Government	□
□	Food distributors	□
□	News media	□

## Results

### Measurement model

The mixed logit model is based on random utility maximization and is a generalization of the traditional multinomial logit model. Compared with the traditional multinomial logit model, the mixed logit model relaxes the strict assumption of independence of irrelevant alternatives, allows coefficients to differ between samples with the same observable characteristics, and does not assume that error terms follow a normal distribution (62, 63). Therefore, the mixed logit model provides a better fit than the traditional multinomial logit model, while highlighting the preferences of different individuals (63). Moreover, it is especially effective when the same respondent is required to make multiple repeated choices (64).

According to the random utility maximization theory, it is assumed that the utility *Unkt* obtained by individual *n* (*n* = 1, 2, …, *n*) from choosing the *kth* (*k* = 1, 2,…, *k*) alternative in situation *t* (*t* = 1, 2, …, *t*) can be divided into two parts. The first part is the observable fixed utility, which depends on the observable attributes of the alternative and individual characteristics. The second part is the unobservable random utility, which represents the effect of unobservable factors on an individual's choice. The utility expression is as follows:


(1) $$\begin{array}{r} Unkt=Vnkt+\varepsilon nkt=\beta nktXnkt+\varepsilon nkt \end{array}$$

where *Unkt* is the utility obtained by individual *n* from choosing *k* in situation *t*; *Vnkt* is the observable utility; *Xnkt* is the attribute variable of actor *k*, which is the observable explanatory vector; and β*n* and ε*nkt* are the random-effect variables that cannot be directly measured.

In the BWS study, respondents were asked to choose the pair of actors with the largest utility difference, i.e., the actors that should bear the greatest and least responsibility. For example, assuming that respondents chose actors *i* and *j* to bear the greatest and least responsibility, respectively, the utility difference between *Unit* and *Unjt* is then greater than that of all other choice sets *M*, where *M* = *K*(*K*−1)−1. By assuming ε*nkt* follows an independent and identically distributed type I extreme-value distribution, the following choice probability of the conditional logit model can be derived:


(2) $$\begin{array}{r} P\left(i\text{,}j\right)=\frac{\exp\left(Vnit-Vnjt\right)}{\sum_{l=1}^{K}\sum_{m=1}^{K}\exp\left(Vnlt-Vnmt\right)-K} \end{array}$$

where *i* is the actor chosen to bear the greatest responsibility, and *j* is the actor chosen to bear the least responsibility. When respondents choose a range of actor combinations that should bear the greatest and least responsibility in situation *t*, the choice probability of Equation (2) can be expressed as follows:


(3) $$\begin{array}{r} Ln\left(\beta n\right)=\overset{T}{\underset{t=1}{\prod }}\frac{\exp\left(Vnit-Vnjt\right)}{\sum_{l=1}^{K}\sum_{m=1}^{K}\exp\left(Vnlt-Vnmt\right)-K} \end{array}$$

The mixed logit probability is a weighted average of each logit variable in the estimation of different β values. The weights are given by the density function *f*(β*n*|*b, w*). The choice probability of Equation (3) can then be expressed as


(4) $$\begin{array}{r} Pn=\int Ln\left(\beta n\right)f\left(\beta n|b,w\right)d\beta n \end{array}$$

### Descriptive analysis results

Respondent demographics are shown in Table 2. There were more females than males in the sample, which is consistent with the fact that in China, most household food purchasers are female. Most respondents were married (70.4%), aged 26–35 (47.0%), had a bachelor's degree (32.9%), had a personal annual income of more than 100,000 yuan (30.7%), and had a household size of (41.0%). In terms of occupation, company employees accounted for the highest proportion (49.5%). In addition, 58.0% of the respondents had children under 18 in the household.

**Table 2:** Demographics of respondents.

**Group**	**Sample size (*n*)**	**Proportion (%)**
**Gender**		
Male	158	39.7
Female	240	60.3
**Age (year)**		
18–25	74	18.6
26–35	187	47.0
36–45	104	26.1
46–55	25	6.3
>55	8	2.0
**Marital status**		
Married	280	70.4
Unmarried	118	29.6
**Education**		
Junior high school or lower	37	9.3
High school	92	23.1
Junior college	125	31.4
Bachelor's degree	131	32.9
Master's degree or higher	13	3.3
**Personal annual income (yuan)**		
<36,000	62	15.6
36,000–50,000	88	22.1
50,000–80,000	79	19.8
80,000–100,000	47	11.8
>100,000	122	30.7
**Household size (** ***n*** **)**		
1	4	1.0
2	18	4.5
3	163	41.0
4	118	29.6
5 or more	95	23.9
**Occupation**		
Government or public institution employee	38	9.5
Company employee	197	49.5
Farmer	11	2.8
Self-employed/unemployed/retired	128	32.2
Student/graduate student	24	6.0
**Having children aged under 18 in the household**		
No	167	42.0
Yes	231	58.0

### Mixed logit model analysis results

The assignment of responsibilities to each responsible actor in the food safety social co-governance system for ensuring food safety was performed using the utility code (Table 3).

**Table 3:** Assignment to responsibility variables.

**Variable**	**Assignment**
Food producers	Actor that should bear the greatest responsibility = 1; Actor that should bear the least responsibility−1; No option = 0
Food distributors	Actor that should bear the greatest responsibility = 1; Actor that should bear the least responsibility−1; No option = 0
Food retailers	Actor that should bear the greatest responsibility = 1; Actor that should bear the least responsibility−1; No option = 0
Government	Actor that should bear the greatest responsibility = 1; Actor that should bear the least responsibility−1; No option = 0
Social organizations	Actor that should bear the greatest responsibility = 1; Actor that should bear the least responsibility−1; No option = 0
News media	Actor that should bear the greatest responsibility = 1; Actor that should bear the least responsibility−1; No option = 0
Citizens	Actor that should bear the greatest responsibility = 1; Actor that should bear the least responsibility−1; No option = 0

The mixed logit model estimates are presented in Table 4. The mean and standard deviation of each variable indicate respondents' perception changes and differences in perceptions regarding the different actors responsible for ensuring food safety, respectively. The greater the standard deviation, the greater the heterogeneity of respondents' perceptions. Analysis of the data in Table 4 demonstrated that, in descending order, the responsibility among the seven actors in the food safety social co-governance system went from the government to producers, social organizations, retailers, distributors, news media, and citizens. The government, producers, and social organization were considered to hold an above- average level of responsibility, where the average was defined by equally allocating the responsibilities to the seven actors. In contrast, respondents assigned a below-average level of responsibility to retailers, distributors, news media, and citizens. Among them, citizens were considered the actor that should bear the least responsibility in the co-governance system.

**Table 4:** Mixed logit model estimates.

**Responsible actors**	**Mean**	**Standard deviation**	**Responsibility share (%)**
Food producers	0.521	0.016	20.81
Food distributors	0.032	0.008	9.67
Food retailers	0.297	0.009	11.59
Government	0.813	0.008	28.19
Social organizations	0.385	0.014	13.90
News media	−0.496	0.015	9.30
Citizens	−0.907	0.021	6.54

## Discussion

Among the seven actors of the five types, food producers, distributors, and retailers together constitute a combined actor, namely food producers and traders. The results in Table 4 indicate that food producers and traders should bear 42.07% of the responsibility in the co-governance system, making it the actor that should bear the greatest responsibility for food safety. This conclusion is completely consistent with the requirements of general international rules and current Chinese food safety laws and regulations, as well as the conclusions of Kleter and Harris et al. (65, 66). However, the responsibilities borne by food producers, distributors, and retailers in the whole process from farm to fork are not the same, and can be individually allocated as 20.81, 9.67, and 11.59%, respectively. In other words, producers have the greatest responsibility, followed by retailers, and distributors bear the least responsibility.

The government should bear 28.19% of the responsibility, which is the second greatest share of responsibility after that of food producers and traders. In addition, social organizations and news media should bear 13.90 and 9.30% of responsibility, respectively, which are considerable shares. As explained above, the news media is regarded as an individual actor because most news media outlets in China have some attributes of government organizations despite being different from government regulators; they also have a unique function that is different from those of any other types of social organizations in the co-governance system. However, news media is also a social organization with an independent legal status. In this sense, the responsibility share of all social organizations, including news media, for food safety co-governance, was 13.90%, which is the third greatest share.

In addition, the responsibility share of citizens was 6.54%, which is the smallest share in the co-governance system. This is consistent with the conclusions of Erdem et al. (58) and is related citizens' position at the of the food supply chain. It also coincides with the reality that Chinese citizens have a weak sense of participation. At present, in China, citizens mainly participate in food safety by complaints and passive reporting after the discovery of defective food products. This reflects the fact few channels exist for Chinese citizens to participate in food safety governance in advance.

However, some controversy still exists in the literature regarding whether citizens are the actor with the least responsibility in the food safety social co-governance system. Redmond and Griffith (67) and Van et al. (68) believed that citizens should bear a great responsibility for food safety. Kjaernes et al. (69) even argued that citizens had a greater responsibility than the government. This discrepancy may arise from differences in the sense of citizenship and food safety regulation systems between countries, and it may also be related to differences in the samples studied and the effects of interest demands of respondents on the empirical results (70). Although the present study is based on data obtained from a citizen survey, it is an objective fact that citizens may ignore or shirk their responsibility, thus resulting in impaired objectivity. Therefore, on the one hand, the responsibility of citizens in terms of food safety co-governance should be objectively recognized, but on the other hand, it should not be arbitrarily extended. Appropriate judgments should be made based on the actual situation. The conclusions of this study provide useful guidance for improving China's food safety social co-governance system. This study argues that although it is important for food producers and traders, the government, social organizations, and news media to shoulder their responsibilities and for continuous efforts to be made to improve laws and regulations to protect citizens' rights in food consumption, citizens should no longer be regarded simply as consumers. Specifically, efforts should still be made to encourage citizens to submit complaints and reports when confronted with food safety problems. But more importantly, at the institutional level, more diversified channels should be designed to ensure that citizens participate in the supervision of food safety in production and marketing, discuss government food safety policies, and develop scientific literacy regarding food safety, so that citizens at different levels will have both the willingness and ability to participate in food safety co-governance. This would not only help build a food safety social co-governance system in China, but is also an inherent requirement of exploring democracy. If the government is able to create a favorable social environment for citizen participation and design channels for citizen participation at the institutional level in the future, it will greatly improve their sense of responsibility and promote their participation in food safety social co-governance. Nevertheless, citizens' responsibilities in food safety social co-governance should not be over-exaggerated.

There are limitations in this study. First, the survey for this study is geographically limited to Wuxi and is not nationwide due to time and fund constraints. Moreover, the demographics of the sample do not perfectly match the overall demographics of Wuxi. Future research should expand the survey area to examine the differences in the public's responsibility perception in different areas to make improvement in depth and breadth. Second, there is no clear division of power and responsibility in the entire food supply chain between the government, market, and society. The role of any individual actor has spatial and temporal boundaries, which, however, are relative and limited, leading to the ambiguity of responsibilities. Therefore, there is still some overlap in the functions undertaken by the seven actors identified in this study in ensuring food safety. This is not only a limitation of this study, but also an unbreakable fact that exists in all countries. Third and lastly, some controversy still exists in the literature regarding whether citizens are the actor with the least responsibility in the food safety social co-governance system. As the present study is based on data obtained from a citizen survey, it is possible that the conclusions may suffer impaired objectivity because citizens may consciously ignore or shirk their responsibility due to their own interest demands. Therefore, future research should include a survey of all responsible actors using new methods to allow a more objective assessment of the responsibilities of all responsible actors, including citizens, in ensuring food safety.

## Data availability statement

The raw data supporting the conclusions of this article will be made available by the authors, without undue reservation.

## Ethics statement

The studies involving human participants were reviewed and approved by Jiangnan University. Written informed consent for participation was not required for this study in accordance with the national legislation and the institutional requirements.

## Author contributions

LW conceptualized the study. KQ processed and analyzed the relevant data, as well as wrote the manuscript. XC collected and analyzed the relevant data and revised the manuscript. All authors contributed to the study design, interpretation the results and manuscript revision, and have approved the final manuscript.

## Funding

This study was supported by the National Social Science Fund of China: Research on social co-governance of food safety risks and cross-border cooperative governance mechanism (20&ZD117).

## Conflict of interest

The authors declare that the research was conducted in the absence of any commercial or financial relationships that could be construed as a potential conflict of interest.

## Publisher's note

All claims expressed in this article are solely those of the authors and do not necessarily represent those of their affiliated organizations, or those of the publisher, the editors and the reviewers. Any product that may be evaluated in this article, or claim that may be made by its manufacturer, is not guaranteed or endorsed by the publisher.
